# Adverse tissue reaction to a next-generation bioceramic sealer: a structured literature review and case report

**DOI:** 10.3389/fdmed.2026.1867974

**Published:** 2026-06-26

**Authors:** Saverio Ceraulo, Antonio Barbarisi, Zhong Hao Hu, Ignazio Migliore, Dorina Lauritano, Gianluigi Caccianiga, Francesco Carinci

**Affiliations:** 1 Department of Medicine and Surgery, University of Milano-Bicocca, Monza, Italy; 2 Dental Clinic, Fondazione IRCCS San Gerardo dei Tintori, Monza, Italy; 3 Department of Translational Medicine, University of Ferrara, Ferrara, Italy

**Keywords:** adverse tissue reaction, apical extrusion, bioceramic sealer, endodontic treatment failure, endodontic treatment outcome

## Abstract

**Background:**

Calcium silicate–based (bioceramic) sealers are widely used in endodontics due to their favorable biological properties and high clinical success rates. Although apical extrusion is generally considered well tolerated, cases of adverse tissue reactions have been reported. The aim of this study was to evaluate clinical outcomes associated with bioceramic sealer extrusion and to analyze potential biological complications.

**Materials and methods:**

A structured literature search was conducted in PubMed (MEDLINE), Scopus, and Web of Science databases for studies published between 2016 and 2026. The search strategy combined terms related to bioceramic sealers, root canal treatment, apical extrusion, and tissue reactions. Study selection followed a PRISMA-informed approach. Human clinical studies were included, and data on extrusion rates, clinical outcomes, and adverse reactions were extracted. In addition, a clinical case of persistent periapical inflammation associated with sealer extrusion is presented.

**Results:**

A total of 18 studies were included. Reported extrusion rates ranged from 11.8% to 60%, depending on the obturation technique. Overall success rates varied between 86% and 98% at follow-ups ranging from 12 months to 5 years. Most randomized clinical trials showed no significant association between sealer extrusion and treatment failure. However, rare complications, including foreign body reactions, neurological involvement, and persistent inflammatory lesions, were described, particularly in cases of prolonged tissue exposure or anatomical proximity to critical structures.

**Discussion:**

While bioceramic sealer extrusion is generally compatible with periapical healing, biological tolerance appears to be condition-dependent rather than absolute. Factors such as material volume, local microenvironment, and host response may influence outcomes. The presented case describes a persistent inflammatory lesion potentially associated with extruded material and requiring surgical intervention, emphasizing the importance of careful follow-up and clinical decision-making.

**Conclusions:**

Bioceramic sealer extrusion is usually well tolerated and not a primary determinant of treatment failure. However, persistent lesions beyond 6–9 months or recurrent clinical symptoms should prompt further investigation and possible intervention. Clinicians should adopt a structured monitoring approach and avoid underestimating atypical biological responses.

## 1 Introduction

In recent decades, bioceramic materials have gained a leading role in various fields of medicine and dentistry, with a particular impact on endodontics (1). In this sector, bioceramic sealers have not only improved the performance of conventional root canal therapies but have also expanded the clinical possibilities related to the maintenance of dental elements and the healing of surrounding tissues (2). Their diffusion represents a paradigm shift, to the point that today these materials are considered among the most reliable options for endodontic treatment, by virtue of their peculiar biological and chemical-physical properties (3). Although most of the evidence still derives from observational studies and trials with relatively short follow-ups, the available clinical data indicate that bioceramic sealers allow high periapical healing rates (generally higher than 80%–90% at 12–24 months) and comparable to those obtained with resin sealers, although it is necessary to conduct long-term controlled studies to consolidate these results (4, 5).

Bioceramic materials are biocompatible compounds developed to perform specific functions within the human organism, capable of integrating with tissues or stimulating their regeneration (6). Depending on their characteristics, bioceramics can behave as bioinert, bioactive or biodegradable substances. The former includes materials such as alumina and zirconia, characterized by stability and resistance. Bioactive ceramics, on the other hand, such as bioglass and hydroxyapatite, interact with tissues stimulating their regenerative response, while biodegradable ones (β-tricalcium phosphate, calcium sulphate) are progressively reabsorbed, leaving space for new tissue formation (7, 8). In the endodontic field, many of these materials are now grouped under the term “bioactive endodontic cements”, which includes MTA (Mineral Trioxide Aggregate) and numerous new generation tricalcium-silicate sealers (for example Biodentine, BioRoot RCS, EndoSequence, iRoot), which share the ability to release calcium and silicate ions and promote the deposition of mineralized hard tissue (9).

This classification reflects the versatility of the group, whose application is not limited to dentistry but also extends to tissue engineering and orthopedic surgery (10).

A turning point in endodontics was the introduction of Mineral Trioxide Aggregate (MTA), developed in the early 1990s and clinically popularized around 2000. MTA is a Portland-based cement enriched with bismuth oxide (Bi_2_O_3_) as a radiopacifier, which, when mixed with water, undergoes hydration reactions to form hydrated calcium silicate and calcium hydroxide (11, 12). Subsequent generations of bioceramics sealers have retained the basic principle but refined it through the use of nanometric tricalcium silicate particles and premixed formulations capable of hardening in the presence of moisture in the root canal system (12). This represented a significant advance over the need for manual mixing of traditional sealers. Literature reviews and comparative studies have highlighted how MTA ensures an effective apical seal and a favorable tissue response, proving more biocompatible and less permeable than various traditional retrograde materials (such as amalgam or IRM), to the point of being considered the golden standard for apical filling and retrograde fillings (13, 14).

Bioceramic materials favor interaction with biological tissues, thanks to their chemical similarity to the composition of natural hard tissues such as bone, enamel, and dentin, consisting mainly of apatitic calcium phosphate (10–12, 15). Compared to traditional sealers, which have an antibacterial action limited to the setting phase, bioceramic sealers guarantee a stable and long-lasting alkaline environment (pH up to 12.5 for approximately 30 days), capable of counteracting the survival of resistant microorganisms within the dentinal tubules, such as Enterococcus faecalis (16). Multispecies biofilm experiments have confirmed that tri-calcium-silicate sealers such as Biodentine, BioRoot RCS, and TotalFill BC Sealer are able to significantly reduce bacterial viability and inhibit the formation of mature biofilms for up to 21 days, with performance comparable or superior to that of some resin sealers (17). This characteristic gives them a unique antimicrobial substance, essential for reducing the risk of infectious recurrence.

Bioceramics are generally characterized by favorable biocompatibility and low inflammatory potential under most clinical conditions (11, 12, 15). Cytotoxicity studies on periodontal ligament fibroblasts and human stromal cells have shown that several calcium silicate-based sealers result in significantly higher cell viability than epoxy or zinc oxide-eugenol sealers and can increase the expression of osteogenic markers (alkaline phosphatase, RUNX2, and type I collagen) (18).

Some recent investigations, however, have highlighted that specific bioceramic formulations, particularly if in prolonged contact with gingival fibroblasts, can induce cytotoxicity and damage to the DNA *in vitro*, suggesting that the excellent biocompatibility of these materials is strictly dependent on the dose, exposure time, and anatomical site where the sealer is found (19).

From a clinical and practical point of view, they offer several advantages: sufficient radiopacity, ease of handling thanks to premixed syringes, hydrophilicity that promotes adhesion, insolubility, slight volumetric expansion that reduces the risk of microleakage and the ability to promote the formation of hydroxyapatite and the deposition of hard tissue (20). Standardized evaluations according to the ISO 6876/2012 standard have demonstrated that bioceramic sealers such as BioRoot RCS TotalFill BC Sealer have adequate flow values ​​and a markedly alkaline pH in the first weeks after setting, characteristics that favor the wettability of the canal walls and contribute to the maintenance of an environment hostile to bacterial survival (21).

The chemical reactions underlying the functioning of bioceramic sealers are (20):

1. Hydration reaction: tricalcium silicate (alite) and dicalcium silicate (belite) react with water to generate hydrated calcium silicate gel (C–S–H), which provides mechanical strength, and calcium hydroxide, responsible for increasing pH and the antibacterial effect.
2. Precipitation reaction: calcium hydroxide interacts with phosphates, leading to the formation of hydroxyapatite (Ca₁₀(PO_4_)_6_(OH)_2_), the main constituent of bone and dentin. This process explains the bioactive activity of the material, which promotes tissue regeneration and facilitates integration with the dentin structure.

*In vitro* morphological observations using scanning electron microscopy have shown, for various tricalcium-silicate sealers, the formation of a continuous layer of carbonated apatite and crystalline extensions that penetrate the dentinal tubules, creating a mineralized interface zone between cementum and dentin that contributes to both chemical and micromechanical sealing (22). The small particle size allows for deep intratubular penetration (up to 1,000 µm deep, assessed with confocal microscopy) and intertubular penetration, ensuring a hydraulic seal. This ensures excellent adhesion to dentin but significantly increases the difficulty of retreatment compared to traditional sealers, with residues persisting after mechanical and ultrasonic removal (23).

In addition to these properties, bioceramic sealers also appear to play a role in modulating the inflammatory and pain response. Studies have demonstrated a reduction in the expression of TRPA1 ion channels in odontoblasts, which are normally activated by inflammatory cytokines and implicated in pain transmission and neurogenic inflammation. This translates into a reduction in postoperative pain, a clinically relevant aspect for patient comfort (24, 25).

Comparison with traditional endodontic materials highlights a qualitative breakthrough introduced by bioceramic materials. Gutta-percha, despite being the standard filling material, does not have bioactive properties nor does it stimulate tissue healing. Zinc oxide-eugenol (ZOE) sealers, on the other hand, exhibit high solubility, long setting times, and volumetric shrinkage, compromising the stability of the seal over time (26, 27). These limitations explain why, despite their historical popularity, traditional materials are now less suited to contemporary clinical needs, while bioceramics offer a combination of biological and mechanical properties more in line with modern endodontic practice (28).

Despite the enthusiasm and numerous supporting studies, the literature contains clinical cases in which bioceramics have not shown the expected behavior. Under particular local conditions, such as the presence of exudate, chronic inflammation, or alterations in the periapical microenvironment, the material's chemical reactions can be compromised and healing hindered. Although these are isolated incidents compared to the majority of positive cases, their relevance should not be underestimated, as they offer food for thought on the real interaction between bioceramic sealers and biological tissues (29).

In light of the increasingly widespread use of these materials in daily practice, any incident that deviates from expectations must be considered an opportunity to delve deeper into the biological mechanisms involved. This paper analyzes a clinical case in which the use of a bioceramic sealer resulted in an adverse tissue response, characterized by persistent inflammation and failed periapical healing. This observation underlines the importance of carefully evaluating not only the strengths, but also the limitations and possible biological variables that influence the behavior of these innovative materials.

## 2 Materials and methods

**Figure 1:**
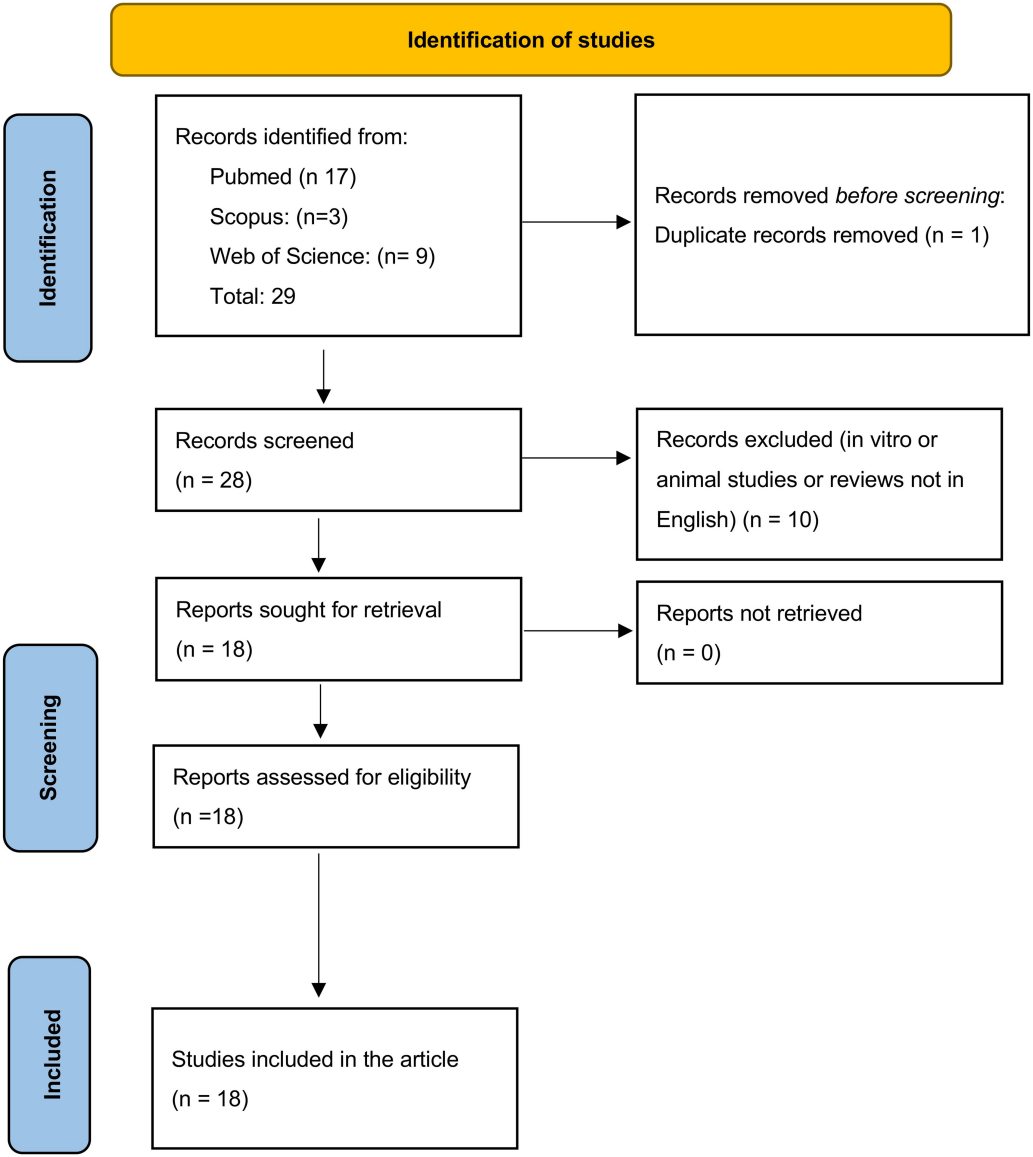
Flowchart of the article selection method according to the PRISMA statement.

An electronic search was conducted in the PubMed (MEDLINE), Scopus and Web of Science database between January 2016 and January 2026. Boolean operators were then used to create a syntax to search for topics as precisely as possible. The review was conducted according to PRISMA guidelines (Figure 1). Although this study does not represent a formal systematic review, a structured search strategy based on PRISMA principles was adopted to improve transparency and reproducibility. The keywords used:

− In Pubmed:
  (
  (“Calcium Silicate Cements"[MeSH]
  OR “Root Canal Filling Materials"[MeSH]
  OR bioceramic sealer*
  OR “calcium silicate-based sealer*”
  OR “hydraulic sealer*”
  OR “MTA-based sealer*”)
  )
  AND
  (
  “Root Canal Therapy”[MeSH]
  OR endodontic treatment
  OR root canal treatment
  )
  AND
  (
  extrusion
  OR “apical extrusion”
  OR overfilling
  )
  AND
  (
  “Foreign-Body Reaction”[MeSH]
  OR “Inflammation”[MeSH]
  OR “Periapical Periodontitis”[MeSH]
  OR “adverse tissue reaction”
  OR “foreign body reaction”
  OR inflammatory response
  OR cytotoxicity
  OR biocompatibility
  )
− In Web of Science:
  “bioceramic sealer” AND “apical extrusion” AND (“foreign body reaction” OR “adverse tissue reaction”)
  “bioceramic sealer” AND “apical extrusion” AND “tissue reaction”
− In Scopus:
  (“bioceramic sealer” OR “calcium silicate-based sealer” OR “hydraulic sealer”) AND (extrusion OR “apical extrusion” OR overfilling) AND (“foreign body reaction” OR “adverse tissue reaction” OR inflammation OR cytotoxicity)

Papers were considered admissible only if they met the following inclusion criteria: studies performed on humans, in English and published within the last 10 years. These are recent studies with the aim of providing as current and reliable as possible impression on the subject.

The exclusion criteria were: not on human studies, not in English, not accessible or available papers and not enough information about the main question of the review.

Titles and abstracts were independently screened by two authors (AB and ZH). If both authors were confident that a study was unsuitable, based on the titles and abstracts, this study was excluded. Any disagreements were resolved by the third author (SC).

Relevant articles were obtained in full-text form, and raw data were extracted independently by each reviewer. Once the research was completed, the results obtained were analyzed and discussed in the following chapters.

### 2.1 Risk of bias assessment

The methodological quality of the included studies was independently assessed by two reviewers. Randomized controlled trials (RCTs) were evaluated using the Cochrane Risk of Bias 2 (RoB 2) tool, considering domains such as randomization process, deviations from intended interventions, missing outcome data, measurement of the outcome, and selection of the reported result. Due to the heterogeneity and limited methodological comparability of the included non-randomized studies and case reports, a formal risk-of-bias assessment was systematically applied only to randomized clinical trials using the Cochrane RoB 2 tool. Case reports and reviews were not included in the risk of bias synthesis due o their inherent methodological limitations.

## 3 Results

There were 29 articles identified after an accurate database searching. Following abstract and full-text reading, a total of 11 articles were excluded according to the exclusion criteria previously established. All reviewed articles selected are shown below (Table 1).

**Table 1:** Articles selected for this work.

N°	Article (Author, Year )	Study design	*n*	Follow-up	Main outcomes	Key conclusions
1	Bardini et al. (30)	RCT	120	4 yrs	Healing 92%; extrusion not detrimental	CS comparable to ZOE
2	Büker et al. (31)	RCT	80	12 mo	Lower early pain; no severe events	CS well tollerated
3	Ensinas et al. (32)	Multicenter RCT	200	24 mo	Extrusion 18%; healing 90%	Extrusion not prognostic
4	Graunaite et al. (33)	RCT	60	12 mo	No difference in pain	Comparable materials
5	Yılmaz & Sarı (34)	RCT	94	12 mo	No significant extrusion impact	Technique-related
6	Martins et al. (35)	Retrospective	180	24 mo	Extrusion not linked to failure	No negative prognosis
7	Kasapoğlu&Doğancalı (36)	Case report	1	6 mo	IAN injury	Risk in anatomical proximity
8	Lee et al. (37)	Clinical study	32	24 mo	Favorable bone healing	Osteoconductive effect
9	Wikström et al. (38)	Longitudinal	52	20 yrs	Stable outcomes	Long-term safety
10	Algar et al. (39)	RCT	100	12 mo	Similar healing rates	CS vs. resin comparable
11	Oliveira et al. (40)	RCT	76	12 mo	Pain influenced by instrumentation	Sealer minor factor
12	Bjørndal et al. (41)	Case report	1	3 yrs	Chronic sinusitis	Severe complication
13	de Figueiredo et al. (42)	RCT	150	24 mo	High success rate	Single-cone effective
14	Musale & Kothare (43)	Case report	1	3 yrs	Healing in immature molar	Favorable response
15	Abada et al. (44)	Prospective RCT	110	12 mo	Technique affects extrusion	Operator-dependent
16	Shoukry et al. (45)	RCT	90	6 mo	No severe adverse events	Safe short-term
17	Song et al. (46)	RCT	72	12 mo	Comparable healing	Sealer-based obturation safe
18	Oliveira et al. (47)	RCT	130	24 mo	QoL unaffected	No long-term difference

RCT, randomized controlled trial; CS, calcium silicate-based sealer; ZOE, zinc oxide-eugenol sealer; QoL, quality of life.

### 3.1 Statistical analysis and bias evaluation

Across the included clinical studies, the overall success rate ranged between 86% and 98% at follow-ups from 12 months to 5 years (30–32, 41, 47). The reported extrusion rate varied between 11.8% and 60%, depending on the obturation technique and activation protocol (32, 44). Importantly, randomized clinical trials consistently showed that sealer extrusion did not significantly reduce periapical healing rates at 1–4 years of follow-up (30, 32, 39).

Neurological complications were rare (<1%) and were almost exclusively associated with extrusion into critical anatomical structures, such as the mandibular canal or maxillary sinus (36, 41).

The risk of bias assessment (Table 2) indicated an overall moderate methodological quality of the included studies. Most randomized controlled trials showed a low risk of bias in the randomization process, while some concerns were identified regarding allocation concealment and blinding procedures. Non-randomized studies presented a higher risk of bias due to inherent methodological limitations, including potential selection bias and lack of standardized outcome assessment.

**Table 2:** Risk of bias assessment of included clinical studies (cochrane RoB 2 framework).

No.	Study (Author, Year)	Study design	Randomization process	Deviations from intended interventions	Missing outcome data	Measurement of outcome	Selection of reported results	Overall risk of bias
1	Bardini et al. (30)	RCT	Low	Some concerns	Low	Low	Some concerns	Moderate
2	Büker et al. (31)	RCT	Low	Some concerns	Low	Low	Some concerns	Moderate
3	Ensinas et al. (32)	Multicenter RCT	Low	Low	Low	Low	Low	Low
4	Graunaite et al. (33)	RCT (split-mouth)	Low	Some concerns	Low	Low	Some concerns	Moderate
5	Yılmaz & Sarı (34)	RCT	Low	Some concerns	Low	Low	Some concerns	Moderate
6	Martins et al. (35)	Retrospective clinical study	High	High	Some concerns	Some concerns	High	High
7	Lee et al. (37)	Clinical study	Some concerns	Some concerns	Low	Some concerns	Some concerns	Moderate
8	Wikström et al. (38)	Longitudinal study	Some concerns	Some concerns	Some concerns	Some concerns	Some concerns	Moderate
9	Algar et al. (39)	RCT	Low	Low	Low	Low	Low	Low
10	Oliveira et al. (40)	RCT	Low	Some concerns	Low	Low	Some concerns	Moderate
11	de Figueiredo et al. (42)	RCT	Low	Some concerns	Low	Low	Some concerns	Moderate
12	Abada et al. (44)	Prospective RCT	Low	Some concerns	Low	Low	Some concerns	Moderate
13	Shoukry et al. (45)	RCT	Low	Some concerns	Low	Low	Some concerns	Moderate
14	Song et al. (46)	RCT	Low	Some concerns	Low	Low	Some concerns	Moderate
15	Oliveira et al. (47)	RCT	Low	Low	Low	Low	Low	Low

RCT, randomized controlled trial; RoB 2, Cochrane Risk of Bias 2 tool.

A high degree of heterogeneity was observed across studies, mainly due to differences in follow-up duration, outcome definitions, and methods used to assess sealer extrusion and periapical healing. These factors should be considered when interpreting the overall findings.

Overall, the methodological quality of the included studies was moderate. Among the selected studies, the majority were randomized controlled trials (RCTs), which generally demonstrated a low risk of bias in the randomization process. However, some concerns were frequently identified in domains related to deviations from intended interventions and selective reporting, mainly due to insufficiently described allocation concealment and blinding procedures. Observational and non-randomized studies showed a higher risk of bias, primarily related to selection processes and lack of control groups, which are inherent limitations of these study designs. Case reports and reviews were not included in the risk of bias synthesis due to their descriptive nature and lack of standardized methodological assessment tools. A recurring methodological limitation across studies was the absence of standardized criteria for defining and quantifying sealer extrusion. In addition, follow-up periods varied considerably (ranging from 6 months to 5 years), and radiographic healing assessment was not uniformly calibrated, contributing to overall heterogeneity. Despite these limitations, the consistency of clinical outcomes observed across moderate- to high-quality RCTs supports the overall evidence suggesting that bioceramic sealer extrusion does not significantly impair periapical healing in most cases. However, these findings should be interpreted with caution considering the methodological variability among studies.

### 3.2 Clinical case

**Figure 2:**
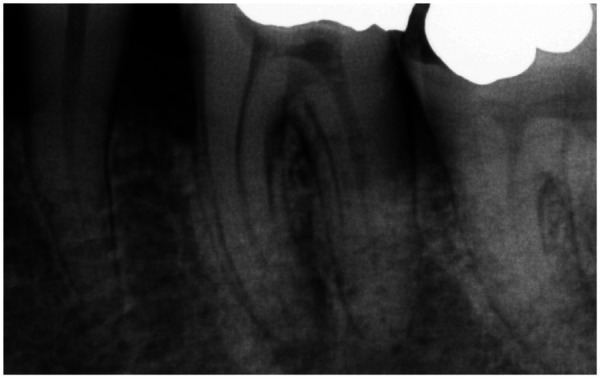
Initial intraoral radiograph of tooth 36.

A 40-year-old woman in good general health presented with no significant medical history, no ongoing medications, and no known allergies. The chief complaint was the presence of a recurrent fistula in the left mandibular region, which had developed following an episode of acute pain consistent with a periapical abscess, with subsequent partial spontaneous symptom resolution. Clinical examination revealed that tooth 36 was intact, without pathological mobility. Periodontal probing showed normal gingival sulcus depth with no evidence of periodontal pockets or attachment loss. Pulp sensibility tests (cold test) were negative, while vertical percussion elicited pain, suggesting apical involvement. Radiographic examination demonstrated a periapical radiolucency affecting both mesial and distal roots (Figure 2). Anatomically, the tooth presented two roots with three canals (two mesial and one distal), consistent with typical mandibular first molar morphology. The clinical case was reported in accordance with CARE reporting recommendations whenever applicable.

#### 3.2.1 Clinical procedure

After access cavity preparation, working length was established, and root canal shaping was performed using a reciprocating system (R25). Irrigation was carried out with 5.25% sodium hypochlorite and 17% EDTA, alternated with saline solution.

Given the presence of apical infection and fistulation, an intracanal medicament based on calcium hydroxide [Ca(OH)_2_]was placed, and the tooth was temporarily sealed. After an adequate period, during which a reduction in clinical signs was observed, the canals were re-accessed, irrigated, and dried with sterile paper points.

**Figure 3:**
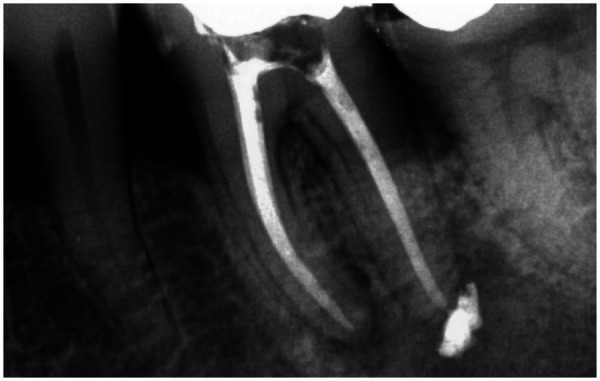
Post-obturation intraoral radiograph of tooth 36.

Obturation was then performed using a single-cone technique with gutta-percha and a premixed hybrid root canal sealer containing calcium silicate components associated with a resin-based matrix. (AH Plus Bioceramic Sealer). A postoperative radiograph revealed apical extrusion of the sealer (Figure 3). The extrusion was unintentional and likely related to the obturation technique and local anatomical conditions. Although extrusion of bioceramic sealers is often considered clinically acceptable due to their bioactive properties, its biological behavior may vary depending on local conditions and the volume of extruded material.

#### 3.2.2 Follow-up

**Figure 4:**
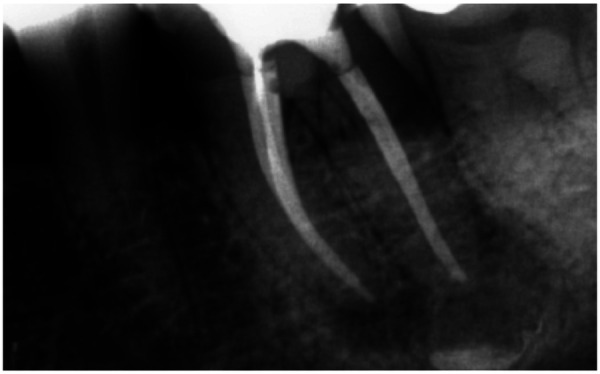
Left post-filling intraoral radiograph of tooth 36.

At the 2-month follow-up, clinical evaluation showed partial regression of the fistula; however, recurrence was observed shortly thereafter, although without significant pain. Radiographic examination revealed persistence of the periapical radiolucency, with radiopaque material consistent with extruded sealer, appearing well demarcated and associated with a possible early cystic transformation (Figure 4). Given the persistence of the lesion, a non-surgical retreatment approach was attempted. The obturation material was partially removed, particularly from the distal canal, where incomplete setting of the sealer was suspected. Calcium hydroxide was reintroduced as an intracanal medicament, and the tooth was temporarily sealed. A transient clinical improvement was observed.

**Figure 5:**
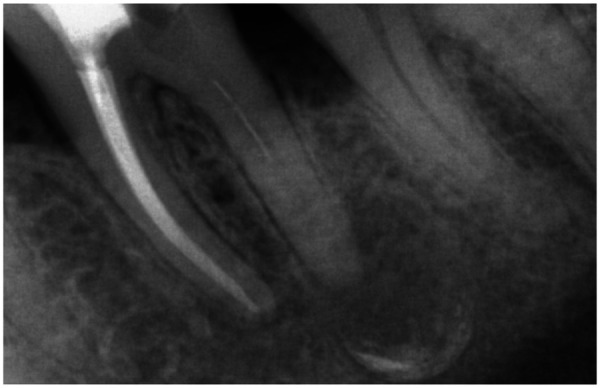
Right post-filling intraoral radiograph of tooth 36.

Approximately three months later, the patient returned with swelling of the left hemiface and recurrence of the fistula. Radiographic findings confirmed persistence of the periapical lesion and the presence of extruded material (Figure 5). In light of the persistent symptoms and lack of resolution following orthograde retreatment, a surgical approach was considered.

Due to the extent of the lesion and the unfavorable clinical evolution, extraction of tooth 36 was performed, followed by surgical curettage of the periapical tissues. Intraoperatively, the lesion appeared as poorly vascularized granulomatous tissue, with foreign material adherent to the surrounding bone.

Histological analysis was not performed in this case, representing a limitation. However, based on intraoperative findings and existing literature, a chronic foreign body reaction characterized by granulomatous inflammation and multinucleated giant cells can be hypothesized. Overall, the unfavorable outcome in this case is likely multifactorial, potentially involving persistent infection, incomplete disinfection, and the biological response to extruded material, rather than being solely attributable to sealer extrusion.

## 4 Discussion

The present study aimed to contextualize a rare adverse tissue reaction following apical extrusion of a next generation bioceramic sealer within the current body of clinical evidence. Overall, the findings of the literature review confirm that calcium silicate–based sealers are associated with high clinical and radiographic success rates, even in cases of unintentional apical extrusion. Across the randomized clinical trials included in this review, success rates ranged between 86% and 98% at follow-ups from 12 months to 5 years (30–32, 39, 46, 47), with most studies reporting no statistically significant association between sealer extrusion and treatment failure. These findings are consistent across multicenter randomized trials and long-term follow-up studies, which indicate that extrusion, when limited in volume and not involving critical anatomical structures, does not appear to negatively influence periapical healing (30, 32, 39). Apical extrusion is not considered a therapeutic objective but may occur unintentionally during obturation procedures and is often clinically tolerated. However, despite this overall favorable evidence, extrusion cannot be considered a biologically neutral event. Its clinical relevance appears to be multifactorial and influenced by variables such as obturation technique, operator experience, material properties, and local anatomical conditions. Several studies have demonstrated that extrusion rates vary significantly depending on the obturation technique and canal preparation strategy, highlighting that extrusion is not solely material-dependent but also technique-sensitive (40, 44). The biological rationale underlying the apparent tolerance to extrusion is related to the bioactivity of calcium silicate–based materials. Their hydration reaction leads to calcium hydroxide release and subsequent hydroxyapatite formation, potentially promoting mineralized tissue deposition and periapical repair. This osteoconductive potential has been confirmed in both clinical and experimental settings, particularly in regenerative and apexification procedures (37, 38). Nevertheless, increasing evidence suggests that the biological response to extruded material is not universally favorable. From a histopathological perspective, foreign body reactions represent a key mechanism underlying adverse outcomes. These reactions are typically characterized by chronic granulomatous inflammation, involving macrophage activation and the formation of multinucleated giant cells attempting to isolate or degrade the foreign material. When the material cannot be resorbed, fibrous encapsulation and persistent inflammatory infiltrate may develop, potentially impairing healing. Importantly, experimental and *in vitro* data indicate that the biological response to calcium silicate–based sealers is dose- and time-dependent (18, 19). While small quantities of extruded material may be well tolerated and gradually resorbed, larger volumes or prolonged exposure can sustain chronic inflammation. Furthermore, local microenvironmental factors—such as impaired vascularization, persistent infection, or continuous exudation—may alter the material's setting reaction and compromise its biocompatibility (29). Severe complications, although rare, have been documented when extrusion occurs in proximity to critical anatomical structures. Reported cases include inferior alveolar nerve injury and maxillary sinus involvement, sometimes leading to persistent neurological deficits or chronic sinusitis (36, 41, 54). These findings underscore that anatomical location is a key determinant in the clinical significance of extrusion and should always be considered in treatment planning and follow-up. Within this context, the clinical case presented in this study represents a relevant deviation from the expected biological behavior. Unlike the majority of cases described in randomized trials, where extrusion is followed by progressive radiographic healing (30–32, 39), this case demonstrated persistent inflammation, recurrent fistulation, and lack of resolution despite orthograde retreatment and intracanal medication. The intraoperative finding of poorly vascularized granulomatous tissue adherent to bone further supports the hypothesis of a chronic foreign body reaction. It is important to emphasize that the unfavorable outcome observed in this case is likely multifactorial. In addition to the potential biological response to extruded material, factors such as persistent infection, incomplete disinfection, and local tissue conditions may have contributed to treatment failure. Therefore, attributing failure solely to sealer extrusion would represent an oversimplification of a complex biological and clinical scenario. Another relevant consideration is the potential influence of obturation technique. Single-cone hydraulic condensation, although widely used and supported by clinical evidence, may predispose to a greater volume of apically extruded material compared to other techniques (42, 48). While extrusion itself is not consistently associated with failure (35), larger volumes of material may increase the likelihood of an unfavorable host response. A critical limitation in the current literature is the reliance on radiographic criteria alone to assess treatment outcomes. Histological confirmation of periapical tissue response is rarely available in clinical studies, which limits the understanding of the true biological mechanisms involved. The present case highlights this gap, as histological analysis was not performed, representing a limitation but also reflecting common clinical practice. The discrepancy between strong population-level evidence and the occurrence of isolated adverse reactions highlights an important clinical implication: apical extrusion of bioceramic sealers should not be automatically considered benign. While generally well tolerated, extrusion may lead to unfavorable outcomes in specific clinical conditions, particularly when associated with persistent symptoms or lack of radiographic healing over time. In this perspective, clinical decision-making should be guided not only by the presence of extrusion itself, but by the dynamic evolution of the lesion. Persistent radiolucency beyond 6–9 months, especially when associated with fistulation or recurrent symptoms, should raise suspicion of an ongoing pathological process, including the possibility of a foreign body reaction, and may justify further intervention. According to the American Association of Endodontists (AAE) guidelines, persistent periapical lesions following adequate root canal treatment should be carefully evaluated and may require surgical intervention when conservative approaches fail.

In summary, the available evidence supports the overall safety and effectiveness of bioceramic sealers in endodontic treatment, even in the presence of apical extrusion (30–32, 39, 46–53). However, biological tolerance appears to be conditional rather than absolute. The coexistence of high success rates and rare but significant adverse reactions suggests that extrusion is not inherently pathological, but its clinical impact depends on a complex interaction between material properties, treatment factors, and host response. Due to heterogeneity in study design, follow-up duration, obturation techniques, and outcome assessment methods, a quantitative synthesis or meta-analysis was not considered appropriate. References 48–54 were used as supplementary background literature and were not part of the formally included clinical studies analyzed in the review.

Despite the generally favorable outcomes reported in several clinical studies, the current evidence remains heterogeneous and should be interpreted cautiously. Considerable variability exists regarding study design, sample size, follow-up duration, obturation techniques, and radiographic assessment methods. Furthermore, many studies included in the present review presented moderate methodological limitations and lacked standardized criteria for evaluating sealer extrusion and periapical healing.

In addition, while most cases of extrusion appear to be clinically tolerated, adverse reactions including persistent inflammation, delayed healing, and possible foreign body responses have been described in selected cases. Therefore, extrusion should not be considered a desirable clinical outcome or a therapeutic objective. Careful working length control and proper obturation techniques remain essential to minimize the risk of overextension. The currently available evidence does not allow definitive conclusions regarding the long-term biological behavior of extruded bioceramic sealers.

It should also be considered that the material used in the present clinical case, AH Plus Bioceramic Sealer, is not a purely calcium silicate–based sealer but a hybrid formulation containing resin-based components. Therefore, the observed tissue response cannot be exclusively attributed to calcium silicate compounds alone, and the potential contribution of the resinous matrix should also be considered.

### 4.1 Limitations of the study

Despite the clinical relevance of the present findings, several methodological and interpretative limitations must be acknowledged. These limitations concern both the heterogeneity of the reviewed studies and the intrinsic nature of the presented case report. The clinical case presented also lacked histopathological and CBCT confirmation, limiting definitive interpretation of the biological reaction associated with the extruded material. Recognizing these aspects is essential to appropriately contextualize the conclusions and to guide future research directions (Table 3).

**Table 3:** Limitations of the study and future research directions.

**Limitation**	**Future Directions to Address the Limitation**
Heterogeneity of included study designs (RCTs, retrospective studies, case reports)	Conduct large-scale, multicenter randomized clinical trials with standardizedprotocols
Variable follow-up periods (6 months to 5 years)	Implement long-term follow-ups (>5 years) with uniform radiographic evaluationc riteria
Lack of standardizeddefinition and quantification of extrusion	Develop validated radiographic and CBCT-based classification systems for sealer extrusion
Predominantreliance on radiographichealingwithouthistologicalconfirmation	Encourage translational studies correlating radiographic findings with histological outcomes
Moderate risk of bias in severalRCTs (unclearal locationconcealment, limited blinding)	Improve reporting transparency following CONSORT guidelines
Absence of volumetric analysis of extruded material	Incorporate 3D CBCT volumetric quantification in future clinical trials
Single case report without hstological confirmation	Report additionalwell-documented cases with histopathological analysis
Limited understanding of patient-relatedbiologicalvariability	Investigate host immune response variability and local microenvironmental factors

### 4.2 Proposed clinical decision-making flowchart for management of bioceramic sealer extrusion

**Figure 6:**
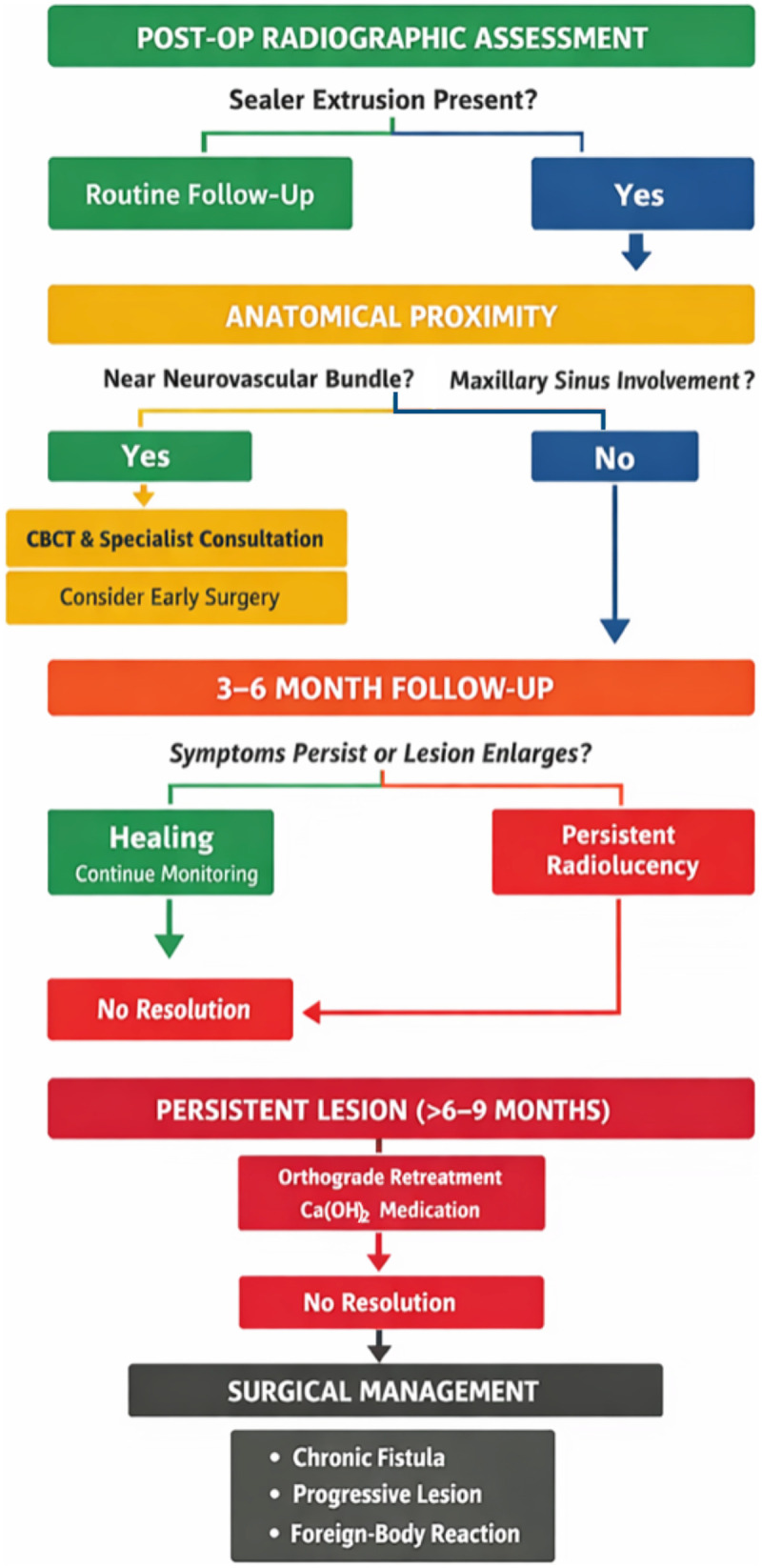
Clinical decision-making flowchart.

The proposed clinical algorithm (Figure 6) is based on the synthesis of current evidence and the findings of the presented case. Although extrusion of calcium silicate-based sealers is generally not associated with unfavorable outcomes (30–32, 39, 48–50), clinical decision-making should not rely solely on the material's biological reputation. The first critical determinant is anatomical proximity. Extrusion involving neurovascular structures or the maxillary sinus has been associated with severe complications, including paresthesia and chronic sinus pathology (36, 41, 54), and therefore requires early imaging and specialist evaluation. In the absence of anatomical risk factors, a conservative monitoring approach is justified. Most randomized trials demonstrate radiographic healing despite extrusion within 12–24 months (30, 32, 39). However, persistent radiolucency beyond 6–9 months, especially when associated with fistulation, should raise suspicion of a foreign-body reaction rather than persistent infection. The present case illustrates that failure to resolve after orthograde retreatment may indicate biologically sustained inflammatory activation. In such scenarios, surgical removal of the extruded material may represent the definitive solution. This structured approach emphasizes that extrusion *per se* is not a prognostic determinant; rather, the persistence of inflammation over time is the critical variable guiding intervention.

## 5 Conclusion

Bioceramic sealers demonstrate high clinical success rates (86%–98%) even in the presence of apical extrusion, and most studies report favorable healing outcomes over time. Extrusion is generally well tolerated and is not, *per se*, a primary determinant of treatment failure. However, biological tolerance is not universal. Rare adverse tissue reactions may occur, particularly in cases of prolonged material exposure, high extrusion volume, or unfavorable local conditions. The present case highlights that persistent inflammatory lesions should not be underestimated and require careful monitoring and, when necessary, timely intervention. Clinicians should adopt a structured follow-up protocol and base clinical decision-making on both radiographic and clinical findings, rather than assuming extrusion to be inherently benign.

## Author's note

This study was conducted in accordance with the Declaration of Helsinki.
